# Microencapsulated OPO Enhances Intestinal SCFA Production by Optimizing Lipid Digestion and Regulating Bile Acid Metabolism in Mice

**DOI:** 10.3390/foods15122075

**Published:** 2026-06-08

**Authors:** Haocheng Liu, Chao Pang, Shiyu Luo, Zhandong Yang, Jiguo Yang

**Affiliations:** 1 School of Public Health and Health Management, Gannan Medical University, Ganzhou 341000, China; ansishc@163.com; 2 School of Food Science and Engineering, South China University of Technology, Guangzhou 510641, China; 3 Guangzhou National Laboratory, Guangzhou 510005, China; yang_zhandong@gzlab.ac.cn

**Keywords:** microencapsulation, OPO structural lipids, short-chain fatty acids, metabolomics, transcriptomics, intestinal digestion

## Abstract

1,3-Dioleoyl-2-palmitoylglycerol (OPO) mimics breast milk fat, yet its regulation of gut short-chain fatty acids (SCFAs) remains unclear. Herein, stable OPO microcapsules (94.12% efficiency) were prepared using soy protein isolate/maltodextrin. Integrating metabolomics and transcriptomics in mice, we found OPO intervention dose-dependently increased acetic, propanoic, and butyric acids in the jejunum and colon. Multi-omics analysis revealed OPO upregulated key genes governing lipid digestion (FABP2, NPC1L1, MTTP) and bile secretion (ABCA1). These results indicate that OPO is closely associated with increased SCFA production, suggesting a potential pathway wherein optimized host nutrient digestion and absorption patterns create a favorable metabolic environment for distal microbial fermentation, rather than merely providing substrates. This study provides correlative multi-omics insights into a potential mechanism for OPO’s health benefits in functional foods.

## 1. Introduction

As one of the three macronutrients, lipids perform physiological functions far beyond mere energy supply. They are critical in maintaining cellular structure, regulating physiological functions, and facilitating the absorption of fat-soluble vitamins [1]. Traditional views often associate fat intake with obesity and cardiovascular diseases, but modern nutrition research indicates that the physiological effects of fats are highly dependent on their molecular structure, particularly the distribution of fatty acids in triglycerides [2,3]. This shift in understanding has propelled functional structured lipids, represented by 1,3-dioleoyl-2-palmitoylglycerol (OPO), into a major research focus.

OPO has garnered significant attention due to the high similarity of its unique molecular structure, palmitic acid at the sn-2 position, and oleic acid at the sn-1 and sn-3 positions, to the core composition of breast milk fat [4]. Extensive research has confirmed that OPO can significantly improve fat and calcium absorption in infants and young children, reduce calcium soap formation, soften stools, and promote intestinal health. For instance, randomized controlled trials have demonstrated that infants fed OPO-enriched formulas exhibit significantly enhanced absorption coefficients for both total fat and calcium compared to those fed standard vegetable oil-based formulas, where palmitic acid is primarily located at the sn-1,3 positions [5]. Mechanistically, this structural advantage prevents the liberation of free palmitic acid in the gut lumen, thereby significantly reducing the formation of insoluble calcium soaps (saponification); consequently, this leads to softer stool consistency and a marked reduction in constipation symptoms comparable to breastfed infants [6]. Furthermore, recent investigations have indicated that OPO supplementation can positively modulate the intestinal environment by promoting the abundance of beneficial Bifidobacterium and Lactobacillus species, suggesting a potential prebiotic-like effect that contributes to overall gut health [7]. However, the commercialization and application of OPO face two major challenges. First, its high content of unsaturated fatty acids makes it prone to oxidation during processing and storage, resulting in poor chemical stability. Crucially, lipid oxidation products (e.g., aldehydes and peroxides) are not only detrimental to flavor but also exhibit cytotoxicity toward gut microbiota, potentially inhibiting their metabolic capacity to produce SCFAs [8,9]. Second, although its advantages in digestion and absorption have been partially elucidated [10,11], the underlying metabolic regulation mechanisms of OPO in vivo remain inadequately explored, particularly its effects on short-chain fatty acids (SCFAs)—key metabolites in the intestinal microenvironment.

Short-chain fatty acids (SCFAs), such as acetic acid, propanoic acid, and butyric acid, are the primary products of dietary fiber fermentation by gut microbiota. They serve not only as an energy source for intestinal epithelial cells but also as crucial signaling molecules that regulate host metabolism and immune function, and the maintenance of intestinal barrier integrity [12]. Disturbance of SCFA levels is closely associated with various metabolic diseases. Current research on how dietary fats influence SCFAs has largely focused on fat quantity or common fatty acid types. Specifically, prolonged consumption of high-fat diets (HFD) has been consistently shown to diminish the abundance of carbohydrate-fermenting genera, such as Bifidobacterium and Roseburia, thereby reducing cecal butyrate levels and exacerbating insulin resistance [13]. Regarding fatty acid types, saturated fatty acids (SFAs) like palmitic acid have been observed to induce distinct microbial dysbiosis and lower SCFA production compared to polyunsaturated fatty acids (PUFAs) [14]. Furthermore, comparative studies have demonstrated that lard-based diets result in significantly lower fecal propionate concentrations and higher inflammatory markers than isocaloric fish-oil-based diets, highlighting the differential regulatory effects of lipid saturation on the gut ecosystem [15]. However, emerging evidence suggests that the impact of dietary lipids on SCFA production extends beyond fatty acid profiles to their stereospecific structure. Modifying the positional distribution of fatty acids on the glycerol backbone significantly alters pancreatic lipase affinity and subsequent bile acid secretion patterns [16,17]. By preventing the formation of insoluble calcium soaps, structured lipids, such as OPO, mitigate intestinal lumen saponification, which would otherwise trap fermentable substrates and disrupt the mucosal microenvironment. This optimized, “cleaner” digestive trajectory thereby enhances the bioavailability of non-lipid substrates for distal microbial fermentation [18,19]. Consequently, this dynamic gives rise to a “structure–digestion–microbiota–metabolism” axis, wherein upstream lipid structural modifications systematically reshape distal gut microbial consortia and their SCFA metabolic outputs [20,21]. Despite this theoretical potential, it remains an underexplored area as to the impact of OPO and other structured lipids with specific space structure on the SCFA profiles across different intestinal segments through the “structure–digestion–microbiota–metabolism” chain, as well as the underlying molecular mechanisms. Microencapsulation technology is an effective strategy to address lipid oxidation stability issues. Selecting appropriate shell materials and processing techniques to encapsulate OPO can significantly extend its shelf life and may regulate its release behavior in the gastrointestinal tract [22,23]; therefore, stabilizing OPO via microencapsulation is a prerequisite for preserving its physiological bioactivity in the gut. However, no study has systematically investigated the effects of microencapsulated OPO on region-specific SCFA production in the intestine, nor the underlying multi-omics mechanisms.

This study aimed to systematically address the aforementioned issues. First, we optimized the preparation process of OPO microcapsules by screening shell materials and drying methods to obtain a product with high encapsulation efficiency and excellent oxidation stability. Subsequently, through animal experiments, we investigated the region-specific and dose-dependent effects of different doses of OPO intervention on SCFA levels in various intestinal segments of mice. Finally, by integrating metabolomics and transcriptomics analysis, we deeply elucidated the molecular mechanisms by which OPO regulated intestinal SCFA production so as to provide solid scientific evidence and theoretical support for the efficient and precise application of OPO in infant formula and functional foods, and the food for special medical purpose (FSMP).

## 2. Materials and Methods

### 2.1. Materials and Reagents

1,3-Dioleoyl-2-palmitoylglycerol (OPO) (content > 90%, with >40% palmitic acid at the sn-2 position) was obtained from Yihai Kerry Arawana Holdings Co., Ltd. (Dongguan, China). Soy protein isolate (SPI) and whey protein isolate (WPI) were both food-grade products purchased from Yihai Kerry Oils & Grains Industries Co., Ltd. (Dongguan, China). Maltodextrin and resistant dextrin were purchased from Baolingbao Biology Co., Ltd. (Dezhou, China). All standards used for short-chain fatty acid (SCFA) analysis (including acetic acid, propanoic acid, butyric acid, isobutyric acid, valeric acid, and isovaleric acid) were HPLC grade (Sigma-Aldrich, St. Louis, MO, USA). All other chemicals and solvents were analytical reagents or chromatography reagents.

### 2.2. Preparation of OPO Microcapsules

OPO microcapsules were prepared using various shell material systems: soy protein isolate with maltodextrin (1:1, *w*/*w*) and soy protein isolate with resistant dextrin (1:1, *w*/*w*). The shell materials were dissolved in deionized water to prepare 10% (*w*/*v*) solutions in which OPO was subsequently added at different core-to-shell ratios (1:1, 1:2, 1:3). The obtained mixture was first pre-emulsified for 10 min at 10,000 rpm using a high-shear disperser (T18, IKA, Staufen im Breisgau, Germany), followed by homogenization for 5 cycles at 35 MPa pressure using a high-pressure homogenizer (JXNANO-15, Shanghai Jingxin, Shanghai, China). The resulting emulsion was spray-dried (ADL311S, Yamato, Tokyo, Japan) or freeze-dried (Alpha1-4Ldphs, Christ, Osterode am Harz, Germany) to obtain microcapsule powder [24]. For freeze-dried samples, the resulting solid matrix was gently milled and passed through a 60-mesh sieve to ensure a uniform particle size distribution (<250 μm) prior to characterization and animal dietary intervention.

The surface oil content and encapsulation rate of these powders were determined using petroleum ether extraction. The powder with the highest encapsulation rate was selected for subsequent analysis, and its acid value and peroxide value were measured according to Chinese National Standard GB 15196-2015 [25] so as to evaluate its oxidation stability.

### 2.3. Animal Experiments and SCFA Analysis

#### 2.3.1. Animal Design and Daily Ration

Twenty-four three-week-old male C57BL/6J mice were obtained from Guangdong GemPharmatech Co., Ltd. (Guangzhou, China) (Reg. No. SCXK (Yue) 2023-0067). All experimental procedures were approved by the Animal Ethics Committee of the Institute of Biological and Medical Engineering, Guangdong Academy of Sciences (Approval No. SYXK-20230183; Date: 26 February 2024) and conducted in strict accordance with the committee’s animal welfare guidelines (Ethics No. K2024-02-026). All animal experiments were conducted in accordance with the ARRIVE guidelines. The sample size was determined based on previous studies investigating the effects of dietary lipids on intestinal SCFA profiles in rodents and practical feasibility constraints. A group size of *n* = 8 was selected to ensure adequate power for detecting biologically meaningful differences in SCFA concentrations while adhering to the 3Rs principle of minimizing animal usage. No formal a priori power analysis was performed.

Mice were housed in a controlled environment with a temperature of 25 ± 2 °C, relative humidity of 60 ± 5%, and a 12 h light/12 h dark cycle, with ad libitum access to food and water. Mice were housed in standard individually ventilated cages (IVC) with sterilized wood-chip bedding. Environmental enrichment was provided in the form of nesting material (cotton squares) and a transparent red plastic shelter in each cage.

Following a one-week acclimatization period on a standard chow diet, the mice were randomly assigned to three groups (*n* = 8): the control group (CON) fed standard rodent chow (supplemented with equivalent amounts of maltodextrin and soy protein isolate used in the microcapsules to account for the wall material intake), the low-dose OPO group (LOPO, daily ration containing 2.25% OPO), and the high-dose OPO group (HOPO, daily ration containing 9% OPO). Randomization was performed using a computer-generated random number sequence (Microsoft Excel RAND function) to allocate animals into the respective groups. To minimize potential confounding effects, cages were arranged on the same rack shelf, and the positions of cages from different groups were alternated. All experimental procedures and sample collections were performed in a fixed order (CON → LOPO → HOPO) across each time point to ensure consistent handling conditions. The OPO used was an optimized microcapsule product (SPI–maltodextrin shell material, core-to-shell ratio of 1:3, freeze-dried). Microencapsulation was employed as a functional prerequisite to ensure the chemical stability of OPO throughout the intervention, as unencapsulated structured lipids are highly prone to oxidation, which could introduce confounding cytotoxic effects on the gut environment.

The intervention period lasted 4 weeks. Animals were monitored daily for general health status, body weight, food/water intake, and any signs of pain or distress (e.g., hunched posture, piloerection, lethargy). The predefined humane endpoint was a body weight loss exceeding 20% of initial weight or any severe clinical signs compromising welfare. During the 4-week study, no animals reached the humane endpoint, and no unexpected adverse events occurred. No specific interventions for pain relief were required beyond standard husbandry practices. A detailed experimental protocol, including the research question, study design, and analysis plan, was prepared prior to study initiation. However, the protocol was not prospectively registered in an online repository.

#### 2.3.2. Sample Collection

Following a 12 h fast, mice were deeply anesthetized with sodium phenobarbital (100 mg/kg) and sacrificed via cervical dislocation. The contents of its jejunum, ileum, and colon were collected, immediately flash-frozen in liquid nitrogen, and then stored at −80 °C in an ultra-low temperature freezer for subsequent analysis.

Due to the nature of the dietary intervention (OPO-containing chow versus control chow), complete blinding of investigators during daily animal husbandry was not feasible. However, outcome assessment was conducted with partial blinding: the personnel performing SCFA quantification via GC-FID and bioinformatics analysis of metabolomic/transcriptomic data were blinded to group allocation. Sample labels were coded by a researcher not involved in data acquisition.

#### 2.3.3. SCFA Quantitative Analysis

All mice completed the 4-week intervention, and no animals were excluded based on health status or body weight. For SCFA analysis, a subset of five mice per group was randomly selected from the eight animals due to practical limitations in sample processing capacity. No data points were excluded from any analyses. The primary outcome measure for this study was the concentration of short-chain fatty acids (acetic, propanoic, and butyric acids) in colonic contents. Secondary outcomes included SCFA concentrations in jejunal and ileal contents, as well as differential metabolites and gene expression profiles from omics analyses.

SCFA concentrations were quantified via gas chromatography (GC) [26] following a modified ether extraction protocol. Briefly, intestinal content samples (50 mg) were homogenized in 500 μL of saturated NaCl solution and incubated for 30 min to facilitate disintegration. The mixture was acidified with 20 μL of 10% (*v*/*v*) sulfuric acid, vortexed for 30 s, and extracted with 800 μL of analytical-grade diethyl ether. After centrifugation (8000 rpm, 4 °C, 15 min), the supernatant was collected and dehydrated using anhydrous Na_2_SO_4_ to remove residual moisture. The organic phase was filtered through a 0.22 μm membrane into autosampler vials.

Analysis was performed on an Agilent 7890A GC system (Agilent Technologies Inc., Santa Clara, CA, USA) equipped with a Flame Ionization Detector (FID) and a capillary column (Agilent Technologies Inc., Santa Clara, CA, USA). High-purity nitrogen served as the carrier gas at 2.0 mL/min. The column temperature was programmed from 110 °C (held for 1 min) to 150 °C at 5 °C/min (held for 5 min). Injector and detector temperatures were set at 270 °C and 280 °C, respectively. Samples (5 μL) were injected in split mode (10:1). Quantification was based on external standard curves (0.05–0.5 mg/mL) constructed for acetic, propanoic, isobutyric, butyric, isovaleric, and valeric acids (R^2^ > 0.99). The analytical method was rigorously validated for accuracy and precision. The average recovery rates for the analyzed SCFAs ranged from 92.5% to 104.2% by spiking standard solutions into the sample matrix. Furthermore, the relative standard deviations (RSDs) for both intra-day and inter-day reproducibility were less than 5.0%, confirming the high reliability of the quantification method.

### 2.4. Metabolomics and Transcriptomics Analysis

#### 2.4.1. Sample Pretreatment

Ileal tissue was selected for multi-omics analysis to capture host gene expression and tissue metabolite profiles, whereas SCFAs were measured in luminal contents to reflect microbial metabolic output. For metabolomics, the tissue was homogenized in pre-chilled 80% methanol, followed by the extraction of its metabolites via a chloroform–liquid phase separation protocol. The extract was then filtered through a membrane filter and stored at −80 °C for UPLC-MS/MS analysis. For transcriptomics, the total RNA was extracted from the ileum tissue using TRIzol reagent (Invitrogen, Carlsbad, CA, USA), and its quality was also validated.

#### 2.4.2. UPLC-MS/MS Analysis of Metabolomics

Metabolic profiling analysis was performed using a Waters ACQUITY UPLC I-Class system (Waters Corporation, Milford, MA, USA) coupled with a Thermo Q-Exactive Focus mass spectrometer (Thermo Fisher Scientific, Waltham, MA, USA). Chromatographic separation was performed on an ACQUITY UPLC HSS T3 column (2.1 × 100 mm, 1.8 μm) using gradient elution with mobile phases A (aqueous solution containing 0.1% formic acid) and B (acetonitrile containing 0.1% formic acid). Mass spectrometry data were acquired in data-dependent acquisition (DDA) mode under both positive and negative ion modes [27].

The raw data were processed using Compound Discoverer 3.1 software for peak alignment, compound identification, and relative quantification. Differential metabolites were screened based on the criteria of Variable Importance Projection (VIP) > 1.0 in the Partial Least Squares Discriminant Analysis (PLS-DA) model and a False Discovery Rate (FDR)-adjusted *p*-value < 0.05 in the one-way analysis of variance (ANOVA) to account for multiple testing comparisons using the Benjamini–Hochberg procedure.

#### 2.4.3. RNA Sequencing and Bioinformatics Analysis

RNA-seq libraries were constructed for sequencing on the Illumina NovaSeq 6000 platform (Illumina, Inc., San Diego, CA, USA). The genes significantly altered by OPO intervention were identified through differential expression analysis. Gene Ontology (GO) and Kyoto Encyclopedia of Genes and Genomes (KEGG) pathway enrichment analysis were conducted on differentially expressed genes (DEGs) to elucidate their biological functions [28].

### 2.5. Statistical Analysis

Data from animal studies and SCFA analysis were presented as mean ± standard deviation (SD). One-way analysis of variance (ANOVA) was performed using SPSS 25.0 software, and the statistical significance was assessed using LSD post hoc tests. *p*-value < 0.05 was considered statistically significant.

## 3. Results

### 3.1. Effects of Various Shell Material Combinations and Drying Methods on the Properties of OPO Microcapsules

This study systematically evaluated the effects of various shell material combinations, core-to-shell ratios, and drying methods on the structure, encapsulation efficiency, and oxidation stability of OPO microcapsules. As shown in Table 1, shell material composition was a key factor influencing microcapsule performance. When soy protein isolate (SPI) and maltodextrin (MD) were used as composite shell materials, the microcapsules exhibited optimal comprehensive performance under a core-to-shell ratio of 1:3 and freeze-drying conditions: 94.12% encapsulation efficiency and surface oil content as low as 0.03 g. SPI possessed excellent interfacial adsorption and film-forming capabilities, stabilizing OPO encapsulation through hydrophobic interactions. MD acted as a filler, filling voids in the protein network during drying. Their synergistic action formed a dense and complete shell material structure that effectively inhibited the migration and exposure of core materials.

The core-to-shell ratio significantly influenced microcapsule structural compactness. In the SPI-MD system, the decrease in core-to-wall ratio from 1:1 to 1:3 markedly increased the encapsulation rate from 76.24% to 94.12%. This indicated that increasing the shell material proportion can facilitate the formation of a more continuous and complete coating layer, thereby reducing structural defects.

The drying method directly influenced the final structure of microcapsules. Compared to spray-drying, freeze-drying was more suitable for encapsulating thermosensitive lipid OPO. Its low-temperature, slow dehydration facilitated uniform solidification of the shell material, preventing surface migration of the core material caused by rapid shrinkage and rupture. Under identical process conditions, freeze-dried samples exhibited an average encapsulation rate 6–8 percentage points higher than spray-dried samples.

The physicochemical stability analysis further validated the effectiveness of microencapsulation. The acid value (0.37–0.38 mg/g) and peroxide value (0.06–0.07 g/100 g) of all samples complied with China’s national food safety standards and exhibited low readings. This demonstrated that successful microencapsulation can effectively isolate oxygen and moisture, fundamentally inhibiting the lipid oxidation chain reaction of OPO and ensuring its long-term stable storage in food systems. In summary, by optimizing the shell material composition (SPI:MD = 1:1), core-to-shell ratio (1:3), and employing freeze-drying technology, OPO microcapsules can be prepared with high encapsulation efficiency, compact structure, and excellent oxidation stability, laying a foundation for subsequent studies in this experiment.

### 3.2. Effects of OPO Intervention on SCFA Levels in Jejunum, Ileum, and Colon of Mice

**Figure 1:**
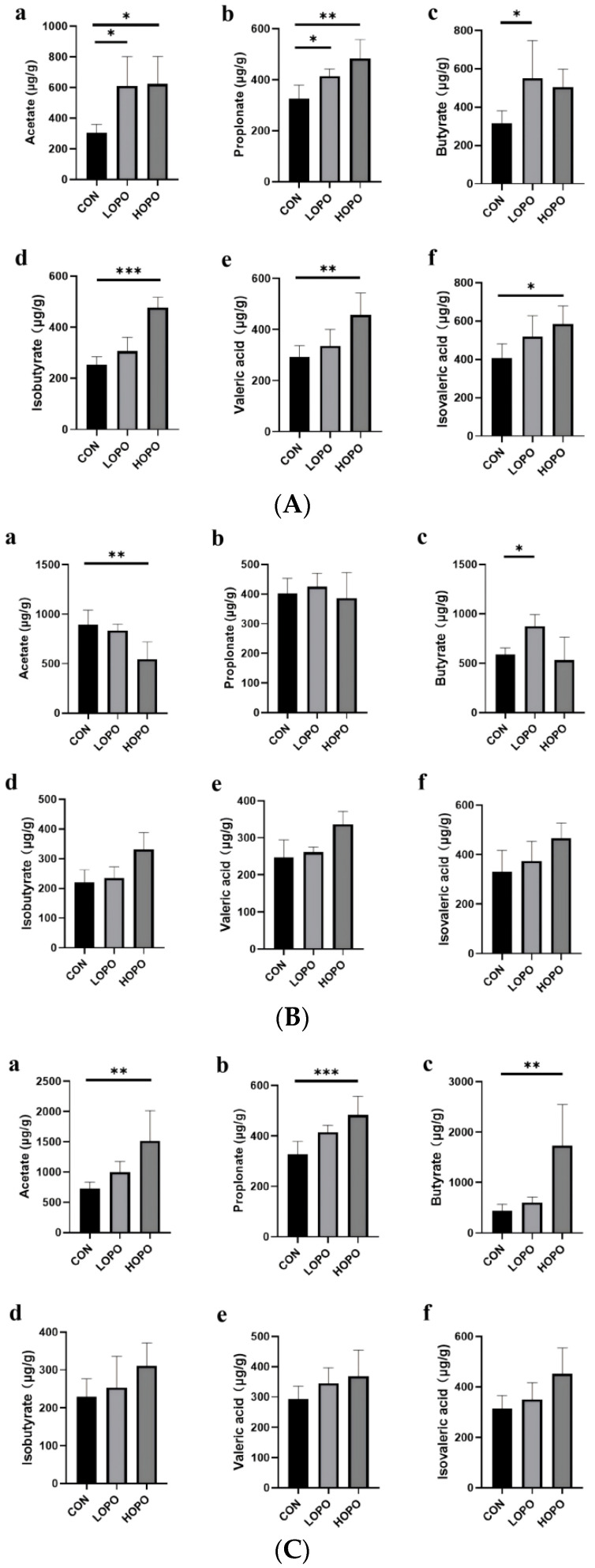
Effects of OPO intervention on main short-chain fatty acid concentrations in mouse jejunum (**A**), mouse ileum (**B**), and mouse colon (**C**). Data are presented as mean ± SD (*n* = 5 per group). Statistical significance was determined using one-way ANOVA followed by LSD post hoc tests. Asterisks indicate significant differences compared to the CON group: * *p* < 0.05, ** *p* < 0.01, *** *p* < 0.001.

To investigate the effects of OPO intervention on SCFA levels in the upper gut of mice, we measured the concentrations of the six major SCFAs (acetic acid, propanoic acid, butyric acid, isobutyric acid, valeric acid, and isovaleric acid) in the jejunal contents of each group (Figure 1A). The results showed that compared with the control group, both LOPO and HOPO groups exhibited significantly increased levels of acetic acid, propanoic acid, butyric acid, and isobutyric acid in the jejunum, with a dose-dependent pattern (LOPO: +102.8%, +29.5%, +80.5%; HOPO: +115.6%, +46.5%, +65.4%). Additionally, the HOPO group exhibited significantly higher levels of isobutyric acid, valeric acid, and isovaleric acid compared to the control group (+86.9%, +69.7%, and +44.5%, respectively), while only isovaleric acid increased in the LOPO group. This indicated that OPO intervention can significantly elevate multiple SCFA levels in the jejunum, with higher doses yielding more pronounced effects.

Compared with jejunum results, the ileum presented higher overall SCFA content, but OPO intervention effects showed distinct patterns (Figure 1B). In the ileum, OPO intervention most significantly influenced the levels of acetic acid and butyric acid. The data indicated that the acetic acid content in the ileum of the LOPO group showed no significant difference compared to the control group, while it significantly decreased by 39.3% in the HOPO group. For butyric acid, they showed a significant increase in the LOPO group compared to the control group, while they exhibited a significant decrease of 44.8% in the HOPO group. No statistically significant differences were observed in propanoic acid, isobutyric acid, valeric acid, and isovaleric acid among the three groups in the ileum. These findings revealed that the impact of OPO intervention on SCFA levels in the ileum differed markedly from that in the jejunum, indicating distinct responses to OPO intervention across different segments of the small intestine.

As the primary site of SCFA production, the colon’s metabolic activity directly reflects the metabolic state following intestinal digestion and absorption. Therefore, we analyzed SCFAs in colonic contents (Figure 1C). The OPO intervention group exhibited significantly increased SCFA levels compared to the control group. Acetic acid levels increased by 38.2% in the LOPO group and 98.6% in the HOPO group compared to the control group. Propanoic acid levels rose 59.3% and 273.7% in the LOPO and HOPO groups, respectively, compared to the control group, demonstrating a clear dose-dependent rise. Butyric acid levels increased by 53.8% in the LOPO group and significantly by 188.7% in the HOPO group. Notably, no significant differences in other SCFAs of the colon were observed across groups, including isobutyric acid, valeric acid, and isovaleric acid. These findings indicated that OPO intervention primarily influenced the production of acetic acid, propanoic acid, and butyric acid in the colon, with higher doses yielding more pronounced effects.

### 3.3. Research of OPO Intervention on Mice Intestinal Metabolomics

#### 3.3.1. Metabolites Classification, Identification, and Differential Analysis

**Figure 2:**
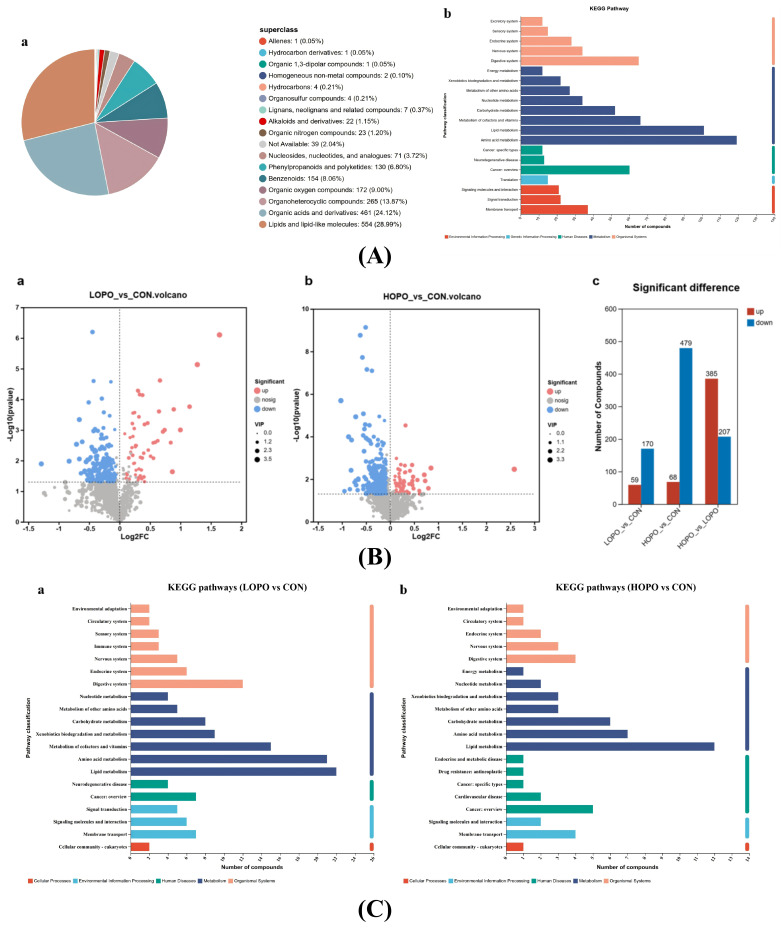
(**A**) Overall characteristics and compound classification of mouse intestinal metabolome after OPO intervention ((**a**) super class; (**b**) KEGG pathway); (**B**) effects of OPO intervention on gut metabolites in mice ((**a**) LOPO VS CON volcano; (**b**) HOPO VS CON volcano; (**c**) significant difference); (**C**) functional enrichment analysis of differential metabolites between OPO intervention groups and control group ((**a**) LOPO VS CON KEGG pathways; (**b**) HOPO VS CON KEGG pathways).

A total of 1852 endogenous metabolites were identified by non-targeted metabolomics analysis (Figure 2(Aa)) of intestinal contents, primarily categorized as lipids and lipoid molecules (28.9%), organic acids and their derivatives (24.1%), and organic heterocyclic compounds (13.9%). KEGG pathway (Figure 2(Ab)) enrichment analysis indicated that these metabolites were extensively involved in the core metabolic pathways of amino acids (150 compounds), lipids (140 compounds), coenzymes and vitamins (90 compounds), nucleotides (70 compounds) and carbohydrates (65 compounds), as well as the functions of digestive system (80 compounds), nervous system (60 compounds), and endocrine (50 compounds) system. These findings indicate that the intestinal metabolites encompass a broad range of biochemical processes.

To reveal the effects of OPO on the expression patterns of the gut metabolite profile of mice, we performed differential analysis of metabolites across groups. As shown in volcano plots in Figure 2(Ba,b), multiple metabolites exhibited significant changes between groups, with the HOPO group showing the most pronounced alterations compared to the CON group. This indicated that high-dose OPO intervention induced more intense metabolic responses. Figure 2(Bc) visually illustrated the distribution of differentially expressed metabolites and dose-dependent effects across groups. It showed that compared to the control group (CON), the low-dose group (LOPO) exhibited 59 significantly upregulated and 170 downregulated metabolites, while the high-dose group (HOPO) showed 68 upregulated and 479 downregulated ones. Compared to LOPO, HOPO showed 385 metabolites upregulated and 207 downregulated. These results indicated that OPO intervention remodeled the gut metabolic profile in a dose-dependent manner.

#### 3.3.2. Functional Enrichment Analysis of Differential Metabolites

To uncover the potential mechanisms by which OPO influenced gut metabolic changes, we performed functional enrichment analysis on differential metabolites. Based on the KEGG database, in the LOPO_vs_CON group (Figure 2(Ca)), differential metabolites were primarily enriched in the metabolism pathways such as lipid metabolism, amino acid metabolism, and carbohydrate metabolism, as well as the environmental information processing pathways, including membrane transport, signaling molecules, and interactions. The pie chart analysis revealed that the differential metabolites primarily comprised lipid and lipoid molecules (36.81%), organic acids and their derivatives (22.54%), organic oxygen compounds (12.72%), and organic heterocyclic compounds (15.93%). Notably, the lipid digestion and absorption pathway showed significant enrichment, involving key metabolites such as cholesterol esters, bile acids, phospholipids, and fatty acids. This suggested that OPO may influence the digestion and absorption process by regulating the metabolites associated with lipid digestion.

In the HOPO_vs_CON group (Figure 2(Cb)), the enrichment pathways for differential metabolites were more extensive and significantly higher, particularly in the pathways such as fat digestion and absorption, protein digestion and absorption, and carbohydrate digestion and absorption, which showed markedly higher enrichment levels compared to the low-dose group. Within these pathways, we observed significant alterations in key digestion-related metabolites, including bile acid components, pancreatic digestive enzyme substrates, and dietary lipid intermediate metabolites. This suggested that high-dose OPO may enhance nutrient digestion efficiency by optimizing digestive enzyme activity and substrate accessibility. The pie chart showed that the proportion of lipid and lipoid molecules further increased in the high-dose group (43.1%), and organic heterocyclic compounds (15.97%), organic acids and their derivatives (15.18%) also accounted for significant proportions. These findings revealed for the first time that OPO may alter the gut microenvironment by optimizing digestion-related metabolism pathways to provide microorganisms with richer fermentation substrates, thereby promoting SCFA production. This offered a mechanistic explanation for the role of the increased SCFAs in the metabolic function enhancement in Section 3.3.

### 3.4. Research of OPO Intervention on Mice Intestinal Transcriptomics

#### 3.4.1. Differential Gene Expression Analysis

**Figure 3:**
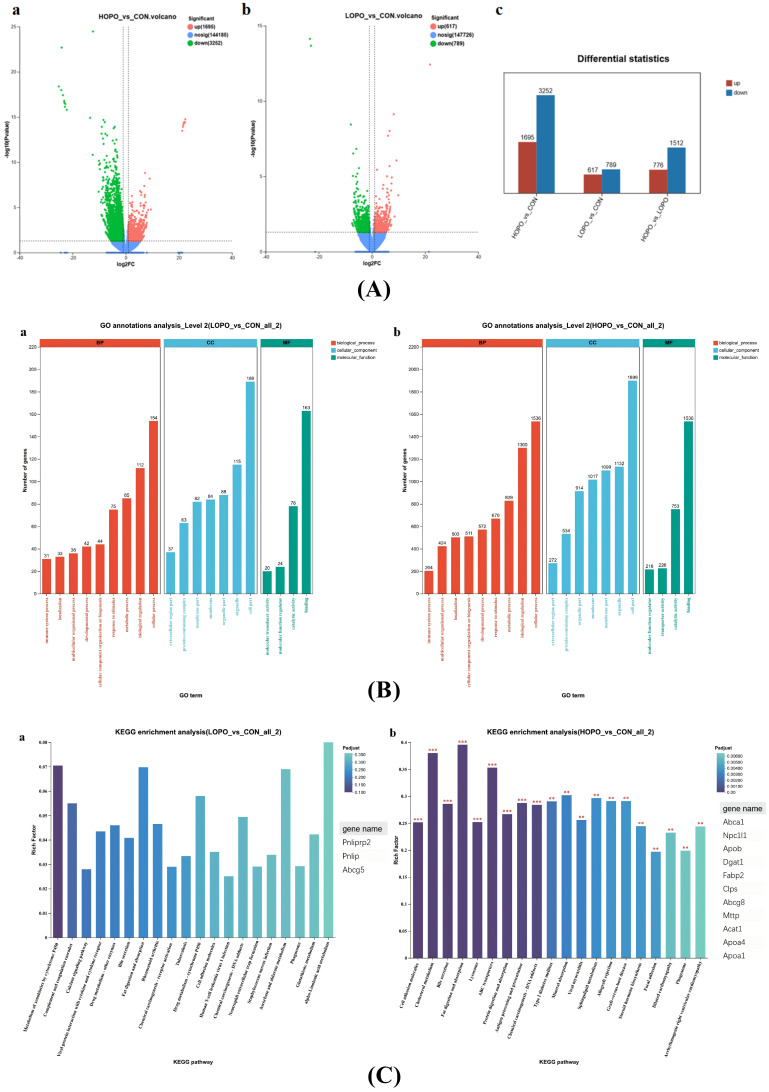
(**A**) Analysis of differentially expressed genes between OPO intervention groups and control group ((**a**) HOPO VS CON volcano; (**b**) LOPO VS CON volcano; (**c**): differential statistics); (**B**) analysis of differentially expressed genes between OPO intervention groups and control group ((**a**) LOPO VS CON volcano; (**b**) HOPO VS CON volcano); (**C**) KEGG pathway enrichment analysis of differentiable expressed genes between OPO intervention groups and control group ((**a**) LOPO VS CON KEGG pathways; (**b**) HOPO VS CON KEGG pathways). For all applicable panels, statistical significance is denoted as ** *p* < 0.01, *** *p* < 0.001.

To investigate the effects of OPO intervention on host gene expression, we performed transcriptome sequencing analysis on the mouse ileum tissue and identified DEGs using the criteria of |log2FoldChange| ≥ 1 and adjusted *p*-value < 0.05 for all comparisons. The volcano plots visually displayed the magnitude and significance of gene expression changes across groups. In the HOPO_vs_CON comparison (Figure 3(Aa)), a total of 4947 DEGs were identified, representing 3.5 times the number of DEGs identified in LOPO vs CON (1406 DEGs). Transcriptomics analysis of ileal tissue further supported the dose-dependent effects of OPO at the gene level (Figure 3(Ab)).

Further GO functional annotation analysis of DEGs elucidated their functional characteristics across three dimensions: biological process (BP), cellular component (CC), and molecular function (MF). The results revealed (Figure 3(Ba)) that DEGs in LOPO_vs_CON were predominantly enriched in multiple critical biological processes, including “cell regulation” (154 genes), “biological regulation” (112 genes), “metabolic processes” (85 genes), “digestion and absorption” (75 genes), and “cell communication” (31 genes). As to cellular components, the DEGs were primarily localized to structures such as “membrane” (189 genes), “organelle” (115 genes), “synapse” (88 genes), and “cytoplasmic part” (84 genes). Regarding molecular functions, DEGs were mainly involved in “catalytic activity” (163 genes), “molecular transduction activity” (78 genes), and “molecular function regulators” (24 genes). GO enrichment results for HOPO_vs_CON DEGs (Figure 3(Bb)) were more abundant and significant. The biological process enrichment entries encompassed “cell regulation processes” (1535 genes), “biological regulation” (1300 genes), “metabolic processes” (829 genes), “digestion and absorption” (670 genes), and “cell communication” (204 genes), with enrichment gene counting 3–5 times higher than the low-dose group. This further reinforced functional enrichment in digestion and absorption, metabolic regulation, and other areas.

#### 3.4.2. KEGG Pathway Analysis of Differentially Expressed Genes (DEGs)

To further elucidate the key signaling pathways and metabolic networks affected by OPO intervention, we performed KEGG pathway enrichment analysis on DEGs. The results (Figure 3(Ca)) revealed that in the LOPO group, genes such as pancreatic lipase (PNLIP, PNLIPRP2) and bile acid transporters (ABCG5/G8) within the “fat digestion and absorption” pathway exhibited significant expression changes. Additionally, alterations in genes within the “calcium signaling” pathway may influence the secretion and activity of digestive enzymes, while the regulation of “cell adhesion molecules” may optimize the absorption function and nutrient transport efficiency of intestinal epithelial cells. These findings revealed the molecular mechanism by which OPO optimized intestinal digestion and absorption functions through regulating the expression of key genes involved in these processes.

The HOPO group exhibited more pronounced and extensive enrichment in digestion and absorption-related pathways (Figure 3(Cb)). The enrichment in digestion and absorption pathways was significantly higher, with highly enriched pathways including “ABC transporters”, “cholesterol metabolism”, “bile secretion”, “fat digestion and absorption”, “protein digestion and absorption”, and “lysosomes”. Specifically, this manifested as coordinated expression changes in cholesterol transport genes (ABCA1, NPC1L1), fatty acid-binding proteins (FABP2), triglyceride transport genes (MTTP), and trypsinogen (PRSS1/2/3). This indicated that OPO did not simply inhibit or promote digestion and absorption but optimized the digestion and absorption to be more efficient and selective by precisely regulating the balance of related gene expression.

#### 3.4.3. Correlation Verification of Key Pathway Genes and SCFA Metabolic Genes

**Figure 4:**
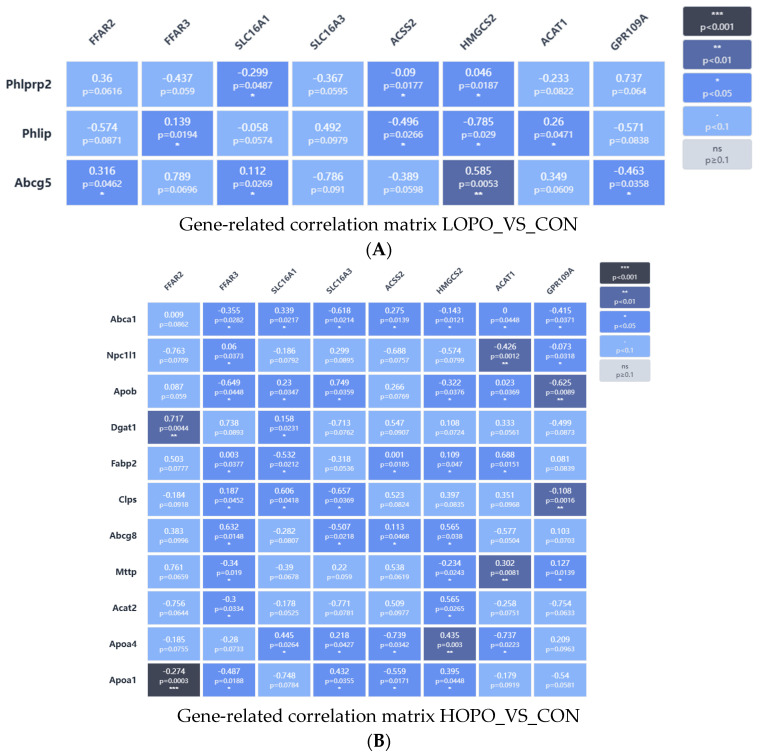
Correlation between key pathway genes regulated by OPO and short-chain fatty acid metabolism genes ((**A**) LOPO VS CON; (**B**) HOPO VS CON). Color intensity represents the correlation coefficient. Statistical significance of the correlations is indicated as follows: * *p* < 0.05, ** *p* < 0.01, *** *p* < 0.001.

Figure 4 displayed the correlation matrix between OPO-regulated upstream metabolism pathway genes and downstream SCFA genes. The results indicated that under the significant regulation of OPO, there were significant correlations between the key genes in the ABC transporter, cholesterol metabolism, and fat digestion and absorption pathways (such as apoa1, Phlip, Abcg5, etc.) and the genes related to short-chain fatty acid metabolism such as FFAR2, FFAR3, and SLC16A3 (especially FFAR2), and the stronger correlations were also observed in the high-dose group. This confirmed that OPO influenced downstream SCFA gene expression by regulating upstream key genes involved in lipid digestion and absorption, thereby promoting the synthesis and accumulation of SCFAs. These findings supported the regulatory pathway, “OPO intake → digestion and absorption optimization → SCFA production increase”, at the level of gene co-expression.

**Figure 5:**
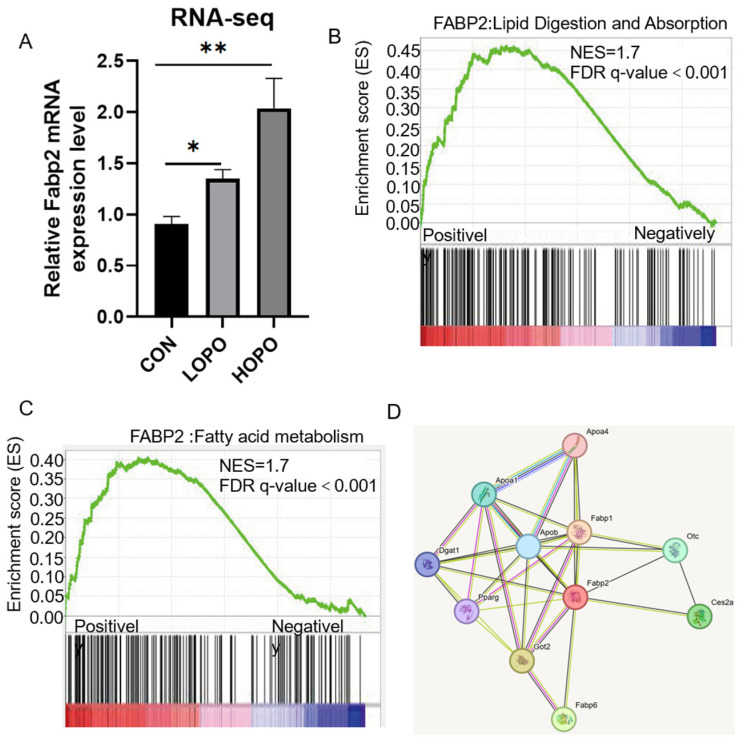
Molecular mechanism analysis of OPO optimizing intestinal fatty acid digestion and absorption through regulating FABP2 gene ((**A**): changes in FABP2 gene expression across different OPO intervention groups; (**B**) the activation status of the FABP2 digestion and absorption pathway; (**C**) the activation status of the FABP2 fatty acid metabolism pathway; (**D**) protein interaction network analysis). In panel (**A**), data are presented as mean ± SD. Statistical significance is denoted as * *p* < 0.05, ** *p* < 0.01.

To further validate the hypothesis that FABP2 acted as a key regulatory factor, we conducted a single-gene analysis of this gene. Transcriptomics sequencing revealed that among the expression changes in FABP2 in various OPO intervention groups, FABP2 expression was significantly upregulated under OPO intervention, exhibiting a pronounced dose-dependent pattern (Figure 5A). Compared to the control group, FABP2 expression was unregulated 1.3-fold in the LOPO group (*p* < 0.05) and 2.0-fold in the HOPO group (*p* < 0.01). Gene Set Enrichment Analysis (GSEA) indicated that under OPO intervention, FABP2-related digestion and absorption pathways (Figure 5B) and fatty acid metabolism pathways (Figure 5C) were significantly enriched, which further confirmed FABP2′s central role in regulating fatty acid metabolism. These findings suggested that OPO synergistically activated transcription programs related to lipid digestion and absorption and fatty acid metabolism by upregulating FABP2 expression. Protein interaction networks (Figure 5D) further revealed direct or indirect interactions between FABP2 and key proteins, including apolipoprotein A1 and A4 (Apoa1, Apoa4), other fatty acid-binding proteins (Fabp1, Fabp6), ornithine transcarbamoyltransferase (Otc), and carbamoyl phosphate synthetase 2 (Cps2). These findings indicated that FABP2 served as a central node in the OPO regulation network, and its upregulation facilitated the coordinated activation of lipid digestion, absorption, and metabolic processes, thereby promoting SCFA production.

## 4. Discussion

This study elucidated the molecular mechanisms by which OPO regulated intestinal metabolism and gene expression after the systematic evaluation of the effects of OPO intervention on SCFA levels in different segments of the mouse intestine, combined with metabolomics and transcriptomics analysis. The results indicated that OPO intervention exhibited significant site-specificity and dose-dependence in SCFA production, and it primarily played roles in multiple pathways, including digestion and absorption optimization, and regulation of microbial composition and metabolic activity.

### 4.1. Encapsulation Performance of OPO Microcapsules

The composite system of soy protein isolate (SPI) and maltodextrin (MD) demonstrated significant advantages in stabilizing OPO, which can be attributed to the complementary behavior of proteins and polysaccharides at the interface. As an amphiphilic protein, SPI possesses excellent surface activity and can rapidly adsorb at the oil–water interface. It forms a viscoelastic interfacial film through unfolding and rearrangement, thereby effectively preventing the coalescence of oil droplets [29]. However, wall films formed solely by SPI are prone to pore formation during the drying process, leading to core leakage. The introduction of the hydrophilic polysaccharide MD as a filler effectively compensated for this defect. Exhibiting good film-forming properties and low oxygen permeability, MD fills the voids within the protein network formed by SPI, enhancing the density of the wall matrix. According to [30], the combination of proteins and maltodextrin can form a denser glassy matrix through hydrogen bonding and electrostatic interactions. This “protein–polysaccharide” complex not only improves the physical stability of the emulsion but also effectively restricts the diffusion of core molecules within the wall matrix, which is consistent with the high encapsulation efficiency (94.12%) observed in this study.

The core-to-wall ratio directly determines the viscosity of the emulsion and the thickness of the wall film. This study showed that as the core-to-wall ratio decreased from 1:1 to 1:3, the encapsulation efficiency of the microcapsules increased significantly. This phenomenon can be explained by the “wall material coverage theory”: at low wall material concentrations (high core-to-wall ratios), there is insufficient film-forming material in the emulsion system to completely cover all oil droplet surfaces. This results in the exposure of some core material on the particle surface after drying, thereby increasing the surface oil content [31]. With an increased proportion of wall material (1:3), the solid content of the continuous phase increases, leading to the formation of a thicker and more continuous wall film upon drying. This effectively reduces cracks and collapse on the particle surface, achieving tight encapsulation of OPO. Similar conclusions were validated by [32] in their study on avocado oil microcapsules, where a higher proportion of wall material contributed to the formation of a protective layer with high mechanical strength, minimizing core loss during processing.

The selection of the drying process is critical for the microencapsulation of heat-sensitive lipids. In this experiment, the encapsulation efficiency of freeze-dried (FD) samples was 6–8 percentage points higher than that of spray-dried (SD) samples, and they exhibited better oxidative stability. This aligns with the findings of [33]. Regarding fish oil microcapsules, the low-temperature environment of freeze-drying effectively inhibited the oxidation of polyunsaturated fatty acids and the generation of off-flavor compounds. Although spray-drying is widely used in industry, its high inlet temperatures often induce rapid water evaporation from droplets, causing a “ballooning effect” [34]. This leads to depressions or even micro-cracks on the particle surface. These microscopic fissures serve as channels for oxygen entry and core leakage, thereby reducing encapsulation efficiency and oxidative stability. In contrast, freeze-drying achieves dehydration through ice crystal sublimation, avoiding heat-induced stress and structural collapse caused by high temperatures. While traditional views suggest that freeze-drying may produce a porous structure, the optimized SPI-MD system allowed the low-temperature solidification process to maintain the original distribution state of the emulsion droplets. The skeletal structure remaining after sublimation was able to retain the core material well, minimizing heat-induced migration of the core to the surface.

Physicochemical stability tests indicated that microencapsulation significantly extended the shelf life of OPO. The dense wall material formed by SPI-MD acted as a physical barrier, blocking direct contact between oxygen, light, and the core material. According to a review by [35], the glass transition temperature (Tg) of protein–polysaccharide wall materials is typically high, keeping them in a glassy state at room temperature. In this state, molecular mobility is extremely low, which greatly limits the diffusion rate of oxygen, thereby kinetically inhibiting the chain reaction of lipid oxidation. The extremely low peroxide values obtained in this study confirmed the effectiveness of this system in protecting high-value structured lipids, providing a theoretical basis for its application in the field of functional foods.

### 4.2. Region-Specific Regulation of SCFAs by OPO Intervention

As crucial signaling molecules, the dynamic variations in SCFAs directly reflect the post-intervention gut metabolic state. The study also indicated that the effects of OPO intervention on intestinal SCFA levels varied across different intestinal segments. In the jejunum and colon, OPO significantly increased the concentrations of major SCFAs such as acetic acid, propionic acid, and butyric acid. Conversely, in the ileum, high-dose OPO reduced levels of certain SCFAs. This region-specific effect suggested that different intestinal segments exhibited distinct physiological response mechanisms to OPO. As the jejunum is the primary site for initial digestion and absorption of nutrients, the increase in its SCFA levels may be attributed to OPO optimization of digestion function in this intestinal segment. He et al. indicated that SCFAs were not only the products of microbial fermentation but also directly participated in the energy metabolism and signal transduction of intestinal epithelial cells, thereby influencing digestion and absorption functions [36]. Elevated SCFAs in the jejunum can provide energy substrates for intestinal epithelial cells while regulating their proliferation and differentiation by activating G protein-coupled receptors (GPR41/43) [37]. Butyric acid, in particular, served as the optimal energy source for intestinal epithelial cells. Its increased concentration was crucial for maintaining intestinal barrier integrity and promoting nutrient absorption. Balogun et al. proved that butyric acid enhanced intestinal barrier function by activating AMP-activated protein kinase (AMPK) and promoting tight junction protein assembly, indicating that this may be a key mechanism through which OPO optimized jejunum function [38].

Unlike the jejunum, OPO intervention resulted in decreased levels of certain SCFAs in the ileum, potentially reflecting OPO’s differential regulation of metabolic functions in the terminal small intestine. Rios-Covian et al. demonstrated significant differences in microbial community composition and fermentation activity across distinct intestinal segments, suggesting that this may be a key factor underlying the site-specific effects of OPO intervention [39]. Another possible explanation is that OPO may promote SCFA absorption in the ileum, leading to decreased detectable levels in the intestinal contents, although this hypothesis requires direct experimental validation. Yuri et al. found that SCFAs can be absorbed by intestinal epithelial cells via monocarboxylate transporter 1 (MCT1) and sodium-coupled monocarboxylate transporter 1 (SMCT1), whose expression and activity varied across intestinal segments [40]. Therefore, OPO intervention may enhance SCFA absorption by modulating the expression or activity of SCFA transporters in the ileum, resulting in reduced detectable levels in the contents. Furthermore, alternative hypotheses must be carefully considered. First, a high lipid load (as administered in the HOPO group) is known to potentially alter gastrointestinal transit time, which could limit the duration available for local microbial fermentation in the terminal ileum. Second, as the ileum is a dynamic transition zone, the concentrated lipid intermediate metabolites might induce a localized microbial shift, temporarily suppressing specific fermentative consortia before the chyme reaches the colon.

The colon was the primary site of SCFA production, and its significantly elevated SCFA levels directly reflected the enhanced metabolic activity within the intestinal microbes. Zhu et al. demonstrated that microbiota community structure and metabolic activity in the colon decisively influenced SCFA production, particularly where the abundance of Ruminococcus, Eubacterium, and Butyrivibrio was closely correlated with butyric acid generation [41]. OPO intervention may promote the growth and metabolic activity of SCFA-producing microbiota by modulating the colonic micro-environment and metabolic status. Furthermore, Li et al. discovered that the distribution of fatty acids within triglycerides can influence their digestion and absorption efficiency in the small intestine so as to alter the quantity and composition of fermentable substrates reaching the colon, thereby regulating SCFA production [42].

### 4.3. Action Mechanism of OPO Revealed by Multi-Omics

Metabolomics analysis revealed that OPO intervention significantly altered the gut metabolite profile, particularly affecting lipids, lipoid molecules, and organic acid metabolites. Differentially expressed metabolites were predominantly enriched in metabolic pathways such as lipid metabolism, amino acid metabolism, and pathways related to the digestive system. The most prominent enrichment occurred in the “fat digestion and absorption” pathway, involving significant alterations in key digestion-related metabolites such as cholesterol esters, bile acids, phospholipids, and fatty acids. These findings aligned with previous studies, such as those of Zhao et al., which demonstrated that structural lipids can significantly alter the hydrolytic patterns and metabolic fates of triglycerides by influencing the spatial arrangement of fatty acids within them [16]. Dong et al. further confirmed that the locations of fatty acids in triglycerides critically influenced pancreatic lipase activity and bile acid secretion, thereby regulating overall lipid digestion efficiency [17]. Kang et al. summarized that different combinations of 2-position fatty acids in triglycerides can significantly affect their digestion and absorption characteristics and physiological functions. Specifically, the structure with medium-chain fatty acids at the 1,3-position can optimize lipid digestion and absorption processes [43]. The high enrichment of digestion system-related pathways and lipid metabolic pathways observed in this study further confirmed that OPO intervention may regulate metabolic networks by optimizing the digestion and absorption process. This provided a metabolic basis for understanding how OPO enhanced SCFA levels.

Transcriptomics analysis further proved the hypothesis that OPO optimized digestion and absorption. KEGG pathway enrichment analysis revealed that OPO intervention significantly influenced the expression of genes associated with pathways including fat digestion and absorption, protein digestion and absorption, bile secretion, and ABC transporters. Notably, the coordinated expression changes in these genes (e.g., ABCA1, NPC1L1, APOB, FABP2, MTTP) indicated that OPO can systematically regulate the gene network governing intestinal lipid digestion, transport, and metabolism to promote efficient and selective lipid utilization rather than merely inhibiting absorption. Xu et al. highlighted that the expression levels and activity of these key lipid metabolism genes determined the efficiency and selectivity of intestinal lipid digestion and absorption, influencing the quantity and composition of undigested lipids entering the colon [44]. This pattern of gene expression change should be interpreted as an optimization rather than a simple inhibition of the digestion and absorption process, and a crucial mechanism by which OPO modulated intestinal functions. Wan et al. discovered that moderate regulation of lipid absorption-related gene expression can prevent excessive absorption and lipid accumulation while maintaining a sufficient supply of substrates available for intestinal microbiota, thereby promoting beneficial bacterial growth and metabolic activity [45]. Larabi et al. further confirmed that alterations in bile acid composition and secretion can indirectly influence SCFA production by regulating the composition and metabolic activity of intestinal microbiota [46]. These studies supported the hypothesis observed in this research that OPO affected SCFA production by regulating the expression of genes involved in digestion and absorption.

### 4.4. Potential Pathways by Which OPO Modulates SCFA Production

The core finding of this study was the link between OPO’s optimization of digestion and absorption and the increase in SCFA production. Multiple studies indicated that intestinal digestion and absorption processes were closely associated with microbial metabolic activity. Here, our multi-omics data provide direct molecular evidence supporting the following three potential pathways. First, unlike standard fats that form insoluble calcium soaps, which trap fermentable nutrients (e.g., dietary fibers) in the chyme, OPO’s unique sn-2 structure prevents saponification. This “cleaner” digestion matrix likely enhances the bioavailability of non-lipid substrates (from the basal diet) for microbial fermentation, despite the high efficiency of lipid absorption upstream. For instance, Cheng and Zhou found that altering lipid digestion rates can influence the amount of undigested lipids reaching the colon, thereby regulating the activity of butyrate-producing bacteria and the production of butyric acid [18]. Wang et al. also confirmed that triglycerides with different structures can modify the composition and availability of substrates accessible to intestinal microbiota by affecting their digestion and absorption patterns, consequently impacting SCFA production. Although direct microbiota profiling was not conducted in the present study, the significant upregulation of host digestion-related genes (e.g., FABP2 and NPC1L1) and the optimization of the metabolic profile suggest a more favorable environment for SCFA-producing bacteria, consistent with previously reported increases in Bifidobacterium and Lactobacillus following OPO intervention. Secondly, OPO may influence intestinal microbial metabolic activity by regulating bile acid composition and secretion [19]. Zhong et al. found that bile acids not only participated in lipid digestion and absorption but also functioned as signaling molecules to modulate intestinal microbial metabolic activity and metabolic activity. OPO intervention may selectively promote the growth of SCFA-producing microbiota by influencing the composition and secretion patterns of bile acids [47]. Mori et al. further confirmed that bile acids regulated intestinal microbial metabolic activity and host metabolism through the farnesoid X receptor (FXR) signaling pathway, which may represent a key mechanism by which OPO influenced SCFA production [48]. Third, the altered metabolic patterns in intestinal epithelial cells induced by OPO intervention may provide a more favorable growth environment for microorganisms. For instance, Shang et al. discovered that the metabolic state of intestinal epithelial cells can indirectly influence anaerobic bacterial activity and SCFA production by regulating oxygen consumption and the local redox environment [49].

### 4.5. Dose-Dependence and Multi-Mechanism Synergistic Investigation

OPO intervention exhibited a pronounced dose-dependent regulation of gut metabolism and gene expression. The high-dose OPO group, with more pronounced functional enrichment, demonstrated significantly higher numbers of differential metabolized compounds and DEGs compared to the low-dose group, indicating the presence of a dose threshold for OPO intervention effects. This finding aligned with the results reported by [50], who observed a pronounced dose-dependent regulation of gut function by structural lipids, suggesting that low doses may be insufficient to induce significant biological effects. The existence of this dose-dependent effect provided an important reference for the clinical application of OPO and the development of functional foods, implying that achieving a certain dose was necessary to obtain optimal outcomes in practical applications.

The high consistency between metabolome and transcriptome enrichment analysis results also provided strong support for the hypothesis that OPO optimized digestion and absorption to enhance SCFA production. Both analyses revealed that OPO intervention significantly influenced pathways including lipid metabolism, cholesterol metabolism, and fat digestion and absorption, and this effect exhibited clear dose dependency. This multi-omics level consistency not only validated the reliability of OPO’s action mechanism but also provided new insights into how OPO synergistically regulates host and microbial metabolism. Nogal et al. confirmed that dietary components, particularly lipid structure, can significantly alter SCFA production patterns and levels by influencing the gut microenvironment and microbial metabolic activity [20]. Liu et al. further demonstrated that structural lipids can increase colonic SCFA production by selectively promoting the growth of SCFA-producing bacteria such as Bacteroides and Ruminococcus [21]. These findings aligned with this study’s observation that OPO elevated SCFA levels by optimizing digestion and absorption. This further supported the complete mechanism chain from OPO intake to the optimization of digestion and absorption, subsequent promotion of SCFA production, and ultimate improvement of metabolic health. Despite these findings, future studies should address the limitations of the current design by incorporating unencapsulated OPO controls and isocaloric diet models to better isolate the specific effects of the encapsulation process and prevent dietary confounding. Additionally, integrating direct microbiota profiling will be crucial to fully validate the microbial consortia responsible for the optimized SCFA production observed. Furthermore, targeted functional validation—such as utilizing in vivo inhibition or knockout models (e.g., FABP2−/− mice)—is required to definitively establish the causal role of the key regulatory nodes identified in our multi-omics analysis.

## 5. Conclusions

Combined with animal experiments and multi-omics technologies, this study elucidated the regulatory mechanism of OPO intervention on the production of short-chain fatty acids (SCFAs) in the gut by systematically optimizing the OPO microencapsulation process. Key findings were as follows: by screening shell material combinations (soy protein isolate–maltodextrin, 1:1, *w*/*w*), optimizing the core-to-shell ratio (1:3), and employing freeze-drying technology, OPO microcapsules were prepared with 94.12% encapsulation efficiency and excellent oxidative stability. Integrated animal experiments and multi-omics analysis revealed that OPO intervention can significantly and specifically increase the levels of beneficial short-chain fatty acid (SCFA) in the jejunum and colon in a dose-dependent manner. OPO dose-dependently upregulated key genes involved in lipid digestion, absorption, and bile secretion (e.g., ABCA1, NPC1L1, FABP2, MTTP), thereby optimizing nutrient utilization. This optimized digestive environment correlates with the provision of favorable substrates for gut microbiota. We propose that OPO potentially provides more suitable fermentation substrates for intestinal microbiota through a suggested “structure–digestion–microbiota–metabolism” chain, thereby ultimately contributing to SCFA production. These findings provide important correlative multi-omics evidence and theoretical insights for the precise application of OPO in functional foods.

## Figures and Tables

**Table 1:** Effects of different wall material systems and drying methods on encapsulation performance of OPO microcapsules.

NO.	Wall Material System	Core-to-Wall Ratio	Drying Method	Surface Oil Content (g)	Total Oil Content (g)	Encapsulation Efficiency (%)
1	Soy Protein Isolate–Maltodextrin (1:1)	1:1	Freeze-drying	0.24	1.01	76.24
2	Soy Protein Isolate–Maltodextrin (1:1)	1:2	Freeze-drying	0.13	0.84	84.52
3	Soy Protein Isolate–Maltodextrin (1:1)	1:3	Freeze-drying	0.03	0.51	94.12
4	Soy Protein Isolate–Maltodextrin (1:1)	1:1	Spray-drying	0.38	1.27	69.87
5	Soy Protein Isolate–Maltodextrin (1:1)	1:2	Spray-drying	0.21	0.87	81.76
6	Soy Protein Isolate–Maltodextrin (1:1)	1:3	Spray-drying	0.08	0.65	87.69
7	Soy Protein Isolate–Resistant Dextrin (1:1)	1:3	Freeze-drying	0.07	0.58	89.66
8	Soy Protein Isolate–Resistant Dextrin (1:1)	1:3	Spray-drying	0.09	0.53	83.02

## Data Availability

The datasets generated and analyzed during the current study are not publicly available due to institutional data storage policies, but are available from the corresponding author on reasonable request.
